# The Impact of Tai Chi on Motor Function, Balance, and Quality of Life in Parkinson's Disease: A Systematic Review and Meta-Analysis

**DOI:** 10.1155/2021/6637612

**Published:** 2021-01-11

**Authors:** Xing Yu, Xinze Wu, Guozhen Hou, Peipei Han, Liying Jiang, Qi Guo

**Affiliations:** ^1^Department of Rehabilitation Medicine, Shanghai University of Medicine and Health Sciences Affiliated Zhoupu Hospital, Shanghai, China; ^2^Department of Rehabilitation Medicine, Shanghai University of Medicine and Health Sciences, Shanghai, China; ^3^College of Exercise and Health Science, Tianjin University of Sport, Tianjin, China

## Abstract

**Objective:**

Parkinson's disease adversely affects function and quality of life, leading to increased mortality. The practice of Tai Chi has been associated with multifaceted improvements in health-related fitness. Considering the limited number of clinical studies included in previous reviews, inconsistent methodological quality, and inconclusive results, this meta-analysis aims to assess the effects of Tai Chi in patients with Parkinson's disease.

**Method:**

Four English language databases and four Chinese databases were systematically searched for existing randomized controlled trials (RCTs) of Tai Chi in Parkinson's disease from database inception through August 1, 2020. Methodological quality was appraised with the Cochrane Risk of Bias tool. A meta-analysis of comparative effects was performed using the Review Manager v.5.3 software.

**Results:**

Seventeen published RCTs totaling 951 subjects were included. Results showed that Tai Chi has a statistically significant effect on the outcomes of gait velocity, unified Parkinson's disease rating scale (UPDRS) motor score, activities-specific balance confidence (ABC) score, and Berg Balance Scale (BBS). The effects on the Timed Up and Go Test (TUGT) and Parkinson's Disease Questionnaire-39 (PDQ-39) were not statistically significant.

**Conclusions:**

This systematic review and meta-analysis of Parkinson's disease and Tai Chi suggests Tai Chi is a relatively safe activity that can result in gains in general motor function and improve bradykinesia and balance. It has no statistically significant advantage for quality of life and functional mobility. Further randomized trials with larger sample sizes and of higher methodological quality are needed to confirm these results and to assess the feasibility of Tai Chi intervention for potential different clinical applications.

## 1. Introduction

Parkinson's disease (PD) is globally the second most common elderly neurodegenerative disease, characterized pathologically by degeneration of dopaminergic neurons in the substantia nigra and the formation of Lewy bodies [1]. Parkinson's patients have gait and posture disorders, resting tremor, bradykinesia, muscle stiffness, and other characteristic motor symptoms [2]. As the disease progresses, patients lose postural stability and the ability to maintain standing balance (including frequent falls), have gait dysfunction, have difficulty managing activities of daily living, and have worsened function and quality of life [3, 4]. Pharmacological treatment is the mainstay of current clinical management. Despite optimal medication, loss of independence, gait dysfunction, and frequent falls persist in patients with PD, which lead to a reduced quality of life and increased mortality [5]. Thus, the Movement Disorder Society Evidence-Based Medicine Panel recommends exercise and physical therapy as an efficacious adjunct to levodopa [6].

Tai Chi is a popular exercise in Chinese society, consisting of slow, gentle, and flowing movements that involve strengthening, balance, postural alignment, mind concentration, relaxation, and breath control [7]. Tai Chi has multifaceted benefits to improving health-related fitness, such as lower extremity muscle strength, balance, flexibility, and cognitive problems [8, 9]. These benefits show potential for improvement of the long-term symptoms of Parkinson's disease [10]. Many studies have previously reported the safety and effectiveness of Tai Chi in Parkinson's disease [10–12].

However, considering the limited number of clinical studies included in previous reviews, inconsistent methodological quality of the published studies, and mixed and inconclusive results, this meta-analysis aims to assess the effects of Tai Chi in patients with Parkinson's disease.

## 2. Methods

This study was designed following the guidelines of the Cochrane Handbook for Systematic Reviews of Interventions and Preferred Reporting Items for Systemic Review and Meta-Analyses (PRISMA) [13].

### 2.1. Search Strategy

The following electronic databases were searched from their inception until 1 August 2020: PubMed, Embase, Cochrane Library, and Web of Science (for relevant English literature) and China National Knowledge Infrastructure (CNKI), Wanfang Database, China Science and Technology Journal (VIP), and Chinese Biomedical Literature Database (CBM) (for relevant Chinese literature). Search strategies were combined as follows: for English databases, free text terms ((Tai Chi) OR (tai ji) OR (tai ji quan) AND (Parkinson's Disease OR Parkinson) were applied and for the Chinese databases, the terms were ((tai ji) OR (tai ji quan) AND pajinsen). Only Chinese and English articles were considered for this meta-analysis. The complete search strategy is shown in the Supplementary Material.

### 2.2. Study Selection

The inclusion criteria for each study were as follows: (1) population consisting of participants with or without Parkinson's disease; (2) using any form of Tai Chi as an intervention; (3) having a control group, other treatments, or another exercise intervention as a comparison group; (4) reporting outcomes of symptoms related to Parkinson's disease or outcomes of exercise training; and (5) using a randomized controlled trial (RCT). Exclusion criteria were as follows: (1) not writing in English/Chinese; (2) having insufficient data or irrelevant outcomes; (3) and having no available full text.

### 2.3. Data Extraction and Quality Assessment

The data were extracted by two independent reviewers (Wu and Hou), who assessed all the studies based on the predesigned standards. Data were checked by a third investigator (Yu). Extracted data from the included studies contained the following information: author name, year of publication, sample size, mean age, interventions (type, duration, and control details), and main outcome measures. We assessed the included studies' quality on the basis of Cochrane Collaboration's risk of bias tool. There were three scores for each item (low risk, unclear, and high risk) according to the following criteria: (1) random sequence generation, (2) allocation concealment, (3) blinding of participants and personnel, (4) incomplete outcome data, (5) selective reporting, and (6) other biases.

### 2.4. Statistical Analysis

All data and statistical analyses were combined and performed using Review Manager v.5.3 software (Cochrane Collaboration, Oxford, UK). The mean difference (MD) with 95% confidence interval (CIs) was used to analyze continuous outcomes. We calculated the mean and standard deviation according to the calculation method mentioned in Cochrane Handbook version 5.1.0, chapter 16.1.3.2: imputing standard deviations for changes from baseline. Heterogeneity across studies was tested with the chi-square test and the Higgins *I*^2^ statistic [14]. The fixed-effects model was applied when statistical heterogeneity was low (*I*^2^ ≤ 50% or Chi^2^test *P* < 0.10); otherwise, a random-effects model was employed. Potential sources of heterogeneity were identified using sensitivity analyses conducted by omitting one study in turn and investigating the influence of a single study on the overall pooled estimate. A *P* value <0.05 was considered statistically significant. Publication bias was detected by Egger's regression asymmetry test.

## 3. Results

### 3.1. Study Selection and the Basic Documents

**Figure 1:**
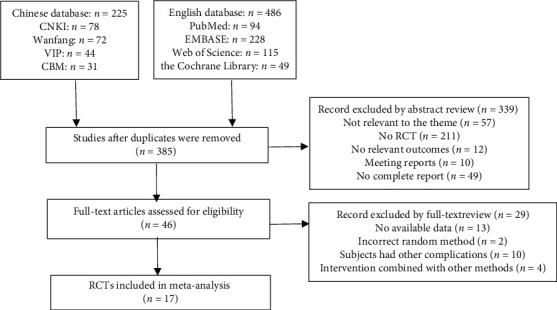
Flow diagram of screening literature studies.

Database searching retrieved a total of 711 studies (486 English literature studies and 225 Chinese literature studies). After removing duplicate entries, 385 studies were screened. 339 studies were excluded based on the abstract and title because of (1) being irrelevant to the theme; (2) no RCT; (3) no relevant outcomes; (4) meeting reports; and (5) no complete report. 46 potentially relevant studies were identified for full-text analysis. 29 were excluded due to (1) no available data; (2) incorrect random method; (3) subjects having other complications; and (4) intervention combined with other methods. After screening, a total of 17 studies were included [4, 11, 12, 15–28]; the flow chart of the study selection is shown in Figure 1. The mean age of the included participants ranged from 53 to 74 years with the intervention duration in the range of 8–24 weeks, exercise time was 40–60 min per session, and the sample sizes varied from 16 to 195. The control groups consisted of usual care, Qi-Gong, Resistance exercise, Stretching, and Routine exercise. The main characteristics of the selected studies are presented in Table 1.

### 3.2. Quality Assessment and Publication Bias

**Figure 2:**
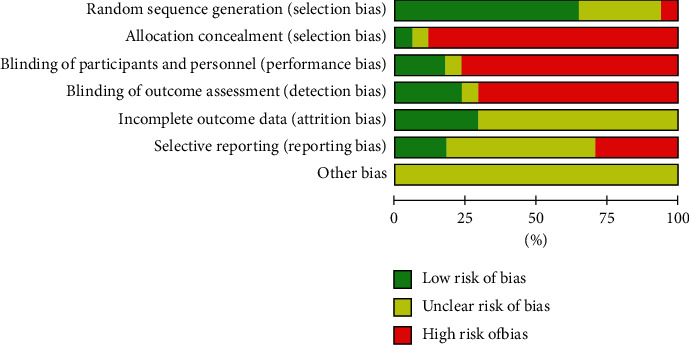
Risk of bias.

**Figure 3:**
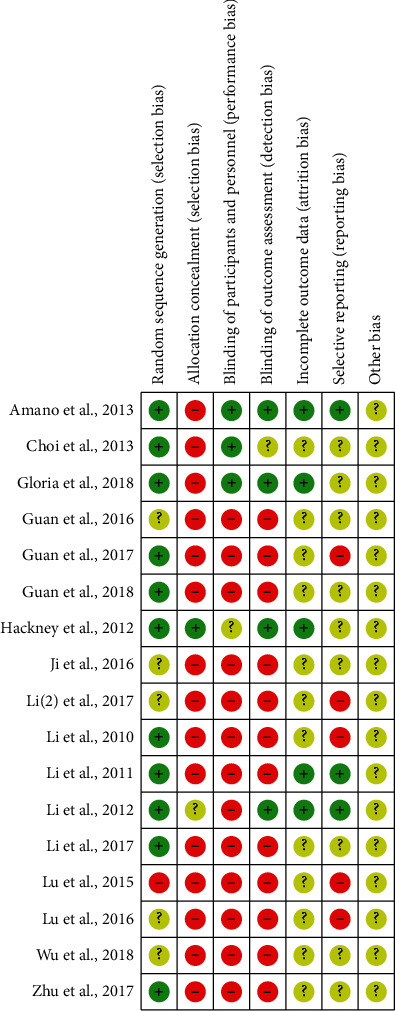
Risk of bias summary.

**Figure 4:**
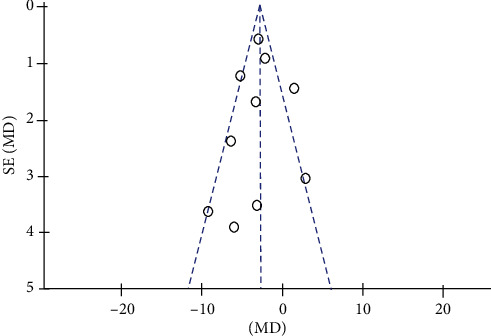
Funnel plot of publication bias.

Figures 2 and 3 show the quality assessment of the included studies using Cochrane Collaboration. Among the 17 RCTs included, 11 studies (65%) [4, 12, 15–19, 21, 23, 24, 27] reported the random sequence generation. One study (5%) [18] reported the use of allocation concealment methods. Five studies (30%) [4, 15–18] described the implementation of blinding of participants and outcome assessment. Twelve studies (80%) [14–19, 21–24, 26] had complete outcome data. Only five studies (30%) [4, 15, 17, 18, 22] reported complete data; others were uncertain. Three studies (17%) [4, 17, 22] described the low risk of selective reports bias; others were uncertain (Figures 2 and 3). No significant publication bias was found from the funnel plot of the 10 studies (Figure 4).

### 3.3. Sensitivity Analysis

Sensitivity analyses were performed based on excluding studies with low quality, small sample sizes, and trials with different control groups to explore potential sources of heterogeneity. For each of these results with high heterogeneity, a separate sensitivity analysis was performed.

### 3.4. Analysis of Outcomes

#### 3.4.1. Gait Velocity

**Figure 5:**
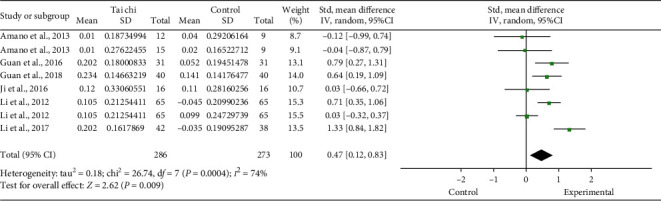
Forest plot of the Tai Chi group versus the control group-gait velocity.

Six studies [4, 11, 17, 19, 23, 25] reported the gait velocity of the Tai Chi group and the control group before and after the trial. Pooled results showed a significant increase of gait velocity in the Tai Chi group after intervention (random-effects model: SMD: 0.47; 95% CI:0.12∼0.83; *P*=0.009; *P* for heterogeneity = 0.0004; *I*^2^ = 74%) (shown in Figure 5). Sensitivity analyses were performed. First, two studies [11, 25] with low quality were excluded. The pooled results showed that this exclusion did not materially alter results and statistical heterogeneity (random-effects model: SMD: 0.48; 95% CI:0.04∼0.92; *P*=0.03; P for heterogeneity = 0. 0002; *I*^2^ = 79%). Second, one study [17] with a small sample size was excluded, but this exclusion did not materially alter results and statistical heterogeneity (random-effects model: SMD: 0.66; 95% CI:0.14∼1.17; *P*=0.01; P for heterogeneity = 0.0002; I^2^ = 85%). Finally, one study [4] that did not use usual care as a control group was excluded, and the pooled results showed that it did not materially alter results and statistical heterogeneity (random-effects model: SMD: 0.98; 95% CI:0.30∼1.66; *P*=0.005; P for heterogeneity = 0.04; *I*^2^ = 76%). A total of three sensitivity analyses reached similar results, reflecting that the results presented here are stable.

#### 3.4.2. UPDRS Motor Score

**Figure 6:**
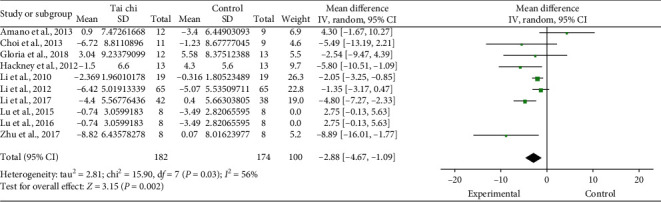
Forest plot of the Tai Chi group versus the control group-UPDRS motor score.

Ten studies [4, 12, 15–18, 22, 23, 26, 27] reported the UPDRS motor score of the Tai Chi group and the control group before and after the trial. The pooled results showed no significant difference between groups after intervention (random-effects model: MD: −1.58; 95% CI: −3.60∼0.43; *P*=0.12; P for heterogeneity <0.001; *I*^2^ = 75%). First, two studies [22, 27] with low quality were excluded. The pooled results showed that it did not materially change statistical heterogeneity, but that overall results changed (random-effects model: MD: −2.88; 95% CI: −4.67∼−1.09; *P*=0.002; *P* for heterogeneity = 0.03; *I*^2^ = 56%). Then, one study [12] with a small sample size was excluded. The pooled results showed this step did not materially alter results and statistical heterogeneity (random-effects model: MD: −2.54; 95%CI: −0.84∼−4.24; *P*=0.003, *P* for heterogeneity = 0.05; *I*^2^ = 53%). Finally, three studies [4, 17, 26] that did not use usual care as a control group were excluded. The pooled results showed that it did not materially alter results but the statistical heterogeneity disappeared (MD: −4.84; 95% CI: −2.83∼–6.85; *P*=0.001, P for heterogeneity = 0.89; *I*^2^ = 0%). Therefore, studies [22, 27] with low quality were excluded from the final results (Figure 6).

#### 3.4.3. PDQ-39 Score

**Figure 7:**
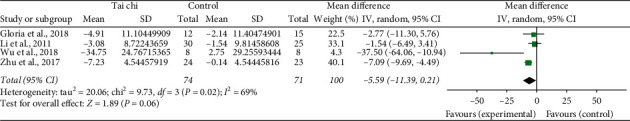
Forest plot of the Tai Chi group versus the control group-PDQ-39.

Four studies [12, 15, 20, 28] reported PDQ-39 score to assess the quality of life. The pooled results showed no significant difference between the Tai Chi group and the control group after intervention (random-effects model: MD: −5.59; 95% CI: −11.39∼0.21; *P*=0.06; P for heterogeneity = 0.02; *I*^2^ = 69%) (Figure 7). After excluding one study [20] with low quality (random-effects model: MD: −4.54; 95% CI: −8.63∼−0.45; *P*=0.03; P for heterogeneity = 0.12; *I*^2^ = 53%) or one study [15] with a small sample size (random-effects model: MD: −4.73; 95% CI: −10.11∼0.64; *P*=0.06; P for heterogeneity = 0.05; *I*^2^ = 74%), the pooled results showed no change in statistical heterogeneity, but results for both changed.

#### 3.4.4. Activities-Specific Balance Confidence

**Figure 8:**
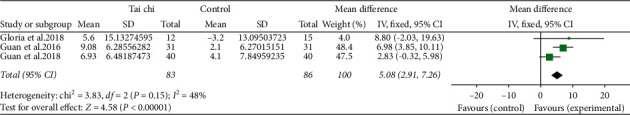
Forest plot of the Tai Chi group versus the control group-activities-specific balance confidence.

Three studies [15,19,25] reported on the activities-specific balance confidence score in the Tai Chi group and the control group before and after the trial. The pooled results showed a significant increase in the Tai Chi group (fixed-effects model: MD: 5.08; 95% CI:2.91∼7.26; *P* < 0.001; P for heterogeneity = 0.15; I^2^ = 48%) (Figure 8).

#### 3.4.5. Timed Up and Go Test

**Figure 9:**
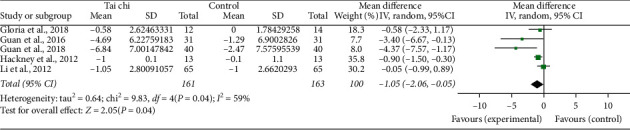
Forest plot of the Tai chi group versus the control group-activities-Timed Up and Go Test.

Five studies [4, 15, 18, 19, 25] reported the Timed Up and Go Test of the two groups before and after the trial. The pooled results showed a significant increase in the Tai Chi group after intervention (random-effects model: MD: −1.05; 95% CI: −2.06∼−0.05; *P*=0.04; P for heterogeneity = 0.04; *I*^2^ = 59%) (Figure 9). First, one study [25] with low quality was excluded, and the pooled results showed that it did not materially alter statistical heterogeneity, but results changed (random-effects model: MD: −0.83; 95% CI: −1.79∼0.14; *P*=0.09; *P* for heterogeneity = 0.06; *I*^2^ = 59%). Second, one study [18] with a small sample size was excluded. The pooled results showed that it did not materially alter results and statistical heterogeneity compared with step 1 (random-effects model: MD: −1.11; 95% CI: −2.97∼0.75; *P*=0.24; P for heterogeneity = 0.04; *I*^2^ = 69%). Finally, one study [4] that did not use usual care as a control group was excluded. The pooled results showed that it did not materially alter results and statistical heterogeneity compared with step 2 (random-effects model: MD: −2.23; 95% CI: −5.91∼1.45; *P*=0.24; P for heterogeneity = 0.04; *I*^2^ = 76%).

#### 3.4.6. Berg Balance Scales

**Figure 10:**
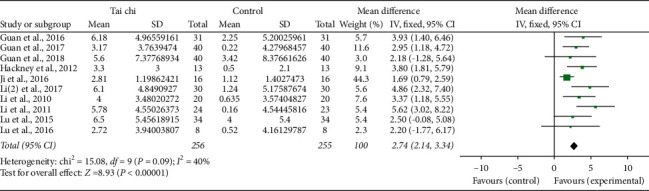
Forest plot of the Tai chi group versus the control group-activities-Berg Balance Scale.

Ten studies [11, 18, 19, 21, 22, 24–28] reported Berg Balance Scale scores in the Tai Chi group and the control group before and after the trial. The pooled results showed significantly increased scores in the Tai Chi group after intervention (random-effects model: MD: 2.74; 95% CI: 2.14∼3.34; *P* < 0.001; *P* for heterogeneity = 0.09; *I*^2^ = 40%) (Figure 10).

## 4. Discussion

Here, we presented a systematic review and meta-analysis that included 17 published RCTs totaling 951 subjects. This work aimed to address the effect of Tai Chi in the management of PD through the evaluation of Tai Chi on motor function, balance, and quality of life. The results provide a new level of evidence for clinical professionals.

### 4.1. Motor Function

The UPDRS motor scale is one of the most commonly used indicators of motor function for Parkinson's disease and was therefore chosen for our meta-analysis to determine the improvement of the general motor function after Tai Chi intervention [1]. Previous studies have shown that the minimal clinically relevant difference (MCRD) of UPDRS III is an improvement of 2.3–2.7 points [29]. The results of this meta-analysis show that the effect of Tai Chi on motor function showed significant improvement in PD patients compared to the control group after excluding studies with low quality (MD: −2.88, *P* < 0.002), which is consistent with previous work [5,10]. After eliminating heterogeneity using a sensitivity analysis, the UPDRS motor scores increased up to 4.84 (MD: −4.84; *P* < 0.001), which surpasses the MCRD. This suggests there is stable evidence that Tai Chi is effective in improving motor function in patients with Parkinson's disease.

Tai Chi is often classified as a low-to-moderate intensity physical activity and is a suitable exercise for individuals in the general population [10, 24] especially those who are middle-aged or older [10, 30]. The Tai Chi protocol stresses weight shifting and ankle sway to effectively improved postural control and walking ability [4]. This focus on balance indicates that Tai Chi would be effective in enhancing neuromuscular rehabilitation and shows that Tai Chi is safe and effective when put in a clinical rehabilitation program.

### 4.2. Balance and Functional Mobility

With the progression of Parkinson's disease, patients have muscle stiffness, lose postural stability, and have gait dysfunction, leading to frequent falls, reduced quality of life, and increased mortality [3–5]. We conducted a meta-analysis of consistent methodological quality of randomized controlled trials and included gait velocity, activities-specific balance confidence (ABC) score, Timed Up and Go Test (TUGT), and Berg Balance Scale (BBS) as indicators to evaluate balance and functional ability.

Many previous studies have reported that the abnormal walking pattern observed in people with PD is characterized by short, shuffling steps [31]. Individuals with PD have a slower gait velocity than those of healthy controls, which is found to be a significant predictor of the first fall [5, 32]. Our meta-analysis demonstrated significant improvement of Tai Chi in gait velocity than in controls (SMD: 0.47; *P*=0.009), and sensitivity analysis stability in the results. Tai Chi improves lower extremity muscle strength, a decisive factor of gait velocity [8]. Thus, an increase in gait velocity may indicate increased muscular strength of the lower limb. Previous studies have also suggested that gait velocity alone may not fully capture the influence of Tai Chi on the Parkinson deficits affecting gait performance [33]. Furthermore, inconsistent results of Tai Chi in improving locomotor control and gait performance have been reported [5, 33]; the clinical application value of Tai Chi in improving the gait velocity needs further research.

Previous studies suggest that BBS and ABC are reliable indicators to measure generic balance performance [34]. Our meta-analysis showed a significant improvement from Tai Chi in BBS (MD: 5.08; *P* ≤ 0.001) and ABC (MD: 2.74; *P* < 0.001) than in controls, consistent with previous work [10]. Sensitivity analysis showed low heterogeneity and stable results. Significantly improved BBS in Tai Chi has been previously reported [10]. These include weight shift, body rotation, slow strides, and single-leg standing in different positions and require delicate joint control with muscle coordination [35]. We augmented these results by adding ABC, which may predict future falls [15, 36], to the meta-analysis of balance function. In general, our results provide stable and homogeneous evidence for Tai Chi in improving the balance ability of Parkinson's patients.

TUGT is a functional mobility balance assessment tool that accurately predicts recurrent falls [37] and is related to the sequencing of several important dynamic stability skills. Our study did not find a stable and reliable result of Tai Chi in improving TUGT for Parkinson's patients (MD: −2.23, *P*=0.24). This is because the studies included in our meta-analysis have high heterogeneity and inconsistent results. The severity of Parkinson's disease may lead to a potential ceiling effect precluding larger magnitude improvements [15]. Therefore, more evidence is needed to explore the effect of Tai Chi on the stability of dynamic balance in patients with Parkinson's disease.

### 4.3. Quality of Life

PD-related quality of life reflects the overall influence of a disease on patients' physical mobility, daily activity, social functioning, psychological wellbeing, and cognition [38]. Stable results were not observed to indicate that Tai Chi could improve the quality of life of patients with Parkinson's disease, which is inconsistent with a previous review report [10]. Previous studies have shown that mind-body exercises such as Tai Chi can have a positive impact through targeting different brain systems that are involved in the regulation of attention, emotion, mood, and executive cognition and improve QOL [9]. Our meta-analysis showed that the results were not stable and highly heterogeneous. While previous studies have shown that Tai Chi can improve gait velocity, it may not fully capture the influence of Tai Chi on Parkinson's deficits affecting the quality of life [39], including gait performance, gait dysfunction, and postural instability [33]. More studies are needed to determine the effect of Tai Chi on the quality of life of Parkinson's patients.

### 4.4. Limitations

This meta-analysis has several limitations. First, the sample sizes of some included RCTs were quite small, and the relatively small number of eligible RCTs in the analysis may lead to limited power and the precision of the findings. Second, blinding of participants or care providers may be difficult in exercise interventions, and the high risk of performance and detection bias might weaken the strength of the evidence. Finally, the diversity of the type and parameters of Tai Chi in the included RCTs also limits the ability to make firm conclusions regarding the specific recommended Tai Chi exercise prescription for each chronic condition.

## 5. Conclusion

In conclusion, this systematic review and meta-analysis article of Parkinson's patients suggests that there exists stable evidence to suggest Tai Chi is a relatively safe program resulting in gains in general motor function and improvements in bradykinesia and balance. However, no statistically significant advantage for quality of life and functional mobility was observed. These results provide new evidence for PD management. Further randomized trials with larger sample sizes and of higher methodological quality are needed to confirm these results and to assess the feasibility of Tai Chi intervention for different clinical application purposes.

## Figures and Tables

**Table 1:** Characteristics of randomized controlled trials included in the meta-analysis.

Study, year	Number of patients (M/F)	Age (T/C)	Study group (n)	Tai Chi group			Control group intervention	Study design
				Outcomes	Duration/exercise time	Frequency/week		
Vergara-Diaz et al., 2018 [15]	32(16/16)	65.7 ± 3.86/62 ± 7.77	T(16)/C(16)	2, 3, 4, 5	6 M/60 min	3	Usual care	2-arm RCT
Choi et al., 2013 [16]	20	60.81 ± 7.6/65.54 ± 6.8	T(11)/C(9)	2	12 W/60 min	3	Usual care	2-arm RCT
Amano et al., 2013 [17]	45(28/17)	P1:64 ± 13/68 ± 7	T(12)/C(9)	1, 2	16 W/60 min	P1: 2	P1 : Qi-Gong	P1:2-arm RCT
		P2:66 ± 11/66 ± 7	T(15)/C(9)			P2: 3	P2 : usual care	P2:2-arm RCT
Li et al., 2012 [4]	195(122/73)	(T)68 ± 9/(R)69 ± 8/(S)69 ± 9	T(65)/R(65)/S(65)	1, 2, 5	24 W/60 min	2	Resistance exercise/Stretching exercise	3-arm RCT
Hackney and Earhart, 2012 [18]	26(21/5)	64.9 ± 8.3/62.6 ± 10.2	T(13)/C(13)	2, 5, 6	10–13 W/60 min	2	Usual care	2-arm RCT
Xihong et al., 2018 [19]	80(44/36)	69.46 ± 5.45/68.61 ± 6.22	T(40)/C(40)	1, 4, 5, 6	24 W/60 min	4	Usual care	2-arm RCT
Tingting et al., 2018 [20]	52(37/15)	62.42 ± 5.37/64.66 ± 5.47	T(30)/C(25)	3	16 W/40 min	4	Routine exercise	2-arm RCT
Guan et al., 2017 [21]	80	No report	T(40)/C(40)	6	24 W/60 min	5	Usual care	2-arm RCT
Li, 2016 [22]	16(10/6)	67.75 ± 6.84/68.20 ± 7.32	T(8)/C(8)	2, 6	8 W/40–60 min	5	Usual care	2-arm RCT
Mingze et al., 2017 [12]	16(5/11)	63.50 ± 6.78/61.50 ± 5.63	T(8)/C(8)	2, 3	24 W/60 min	2	Routine exercise	2-arm RCT
Lin, 2017 [23]	80(44/36)	65.25 ± 6.37/67.78 ± 5.36	T(42)/C(38)	1, 2	16 W/60 min	3	Usual care	2-arm RCT
Li(2) et al. 2017 [24]	60	No report	T(30)/C(30)	6	12 W/60 min	4	Usual care	2-arm RCT
Xihong et al., 2016 [25]	62(33/29)	70.23 ± 4.24/69.71 ± 4.13	T(31)/C(31)	1, 4, 5, 6	12 W/60 min	4	Usual care	2-arm RCT
Ji et al., 2016 [11]	32(17/15)	56.06 ± 11.16/59.13 ± 11.22	T(16)/C(16)	1, 6	3 M/60 min	Not specified	Usual care	2-arm RCT
Yi et al., 2010 [26]	40(23/17)	63.35 ± 8.72/64.83 ± 9.29	T(20)/C(20)	2, 6	3 M/60–90 min	5	Walking exercise	2-arm RC
Li, 2011 [28]	47(22/25)	68.28 ± 6.62/67.13 ± 6.73	T(24)/C(23)	3, 6	8 W/60–90 min	5	Walking exercise	2-arm RCT
Caifeng and Daohonget, 2015 [27]	68(37/31)	67.1 ± 1.5/65.2 ± 1.3	T(34)/C(34)	2, 6	2 M/60–65 min	5	Usual care	2-arm RCT

T, Tai Chi group; C, control group; R, resistance training; S, stretching exercise; outcome 1, gait velocity; outcome 2, unified Parkinson's disease rating scale motor score (UPDRS motor score); outcome 3, Parkinson's Disease Questionnaire-39 (PDQ-39) score; outcome 4, activities-specific balance confidence (ABC) score; outcome 5, Timed Up and Go Test (TUGT); outcome 6, Berg Balance Scale (BBS); M, months; W, weeks.

## Supplementary Materials

Supplementary Materials The complete search strategy is shown in the Supplementary Materials.
