# Supplementary material for: The Impact of Tai Chi on Motor Function, Balance, and Quality of Life in Parkinson's Disease: A Systematic Review and Meta-Analysis
Source: Evid Based Complement Alternat Med. 2021 Jan 11;2021:6637612. doi: 10.1155/2021/6637612 (PMC7814935; doi:10.1155/2021/6637612)
Supplement: Supplementary Materials — The complete search strategy is shown in the Supplementary Materials. [file 6637612.f1.docx]

# The Impact of Tai Chi on Motor Function, Balance and Quality of Life in Parkinson’s Disease: A Systematic Review and Meta-Analysis

Xing Yu, Xinze Wu, Guozhen Hou, Peipei Han, Liying Jiang, and Qi Guo

**Supplementary Material: Search strategy**

**PubMed**

("Tai Chi" OR "tai ji" OR "tai ji quan") AND ("Parkinson's Disease" OR Parkinson)

**Embase**

#1 "Tai Chi" OR "tai ji" OR "tai ji quan"

#2 "Parkinson's Disease" OR Parkinson

#3 #1and #2

**Cochrane Library**

(Parkinson's Disease [MeSH Terms] OR Parkinson) AND (Tai Chi [MeSH Terms] OR tai ji OR tai ji quan) in Title, Abstract, Keywords

**Web of Science**

#1 TS = (Parkinson's Disease OR Parkinson)

#2 TS = (Tai Chi OR tai ji OR tai ji quan)

#3 #2 AND #1

**China National Knowledge Infrastructure (CNKI)**

(SU=帕金森OR SU=帕金森病) AND (SU=太极OR SU=太极拳)

**Wanfang Database**

Title = ((“帕金森” OR “帕金森病”)AND (“太极” OR “太极拳”) or Abstract =((“帕金森” OR “帕金森病”)AND (“太极” OR “太极拳”) or Keywords = ((“帕金森” OR “帕金森病”)AND (“太极” OR “太极拳”) )

**China Science and Technology Journal (VIP)**

KY=（帕金森OR帕金森病）and KY=（太极OR太极拳）

**Chinese Biomedical Literature Database (CBM)**

#1 帕金森[MeSH Terms] OR帕金森病

#2 太极OR太极拳

#3 #1and #2
